# Retinal Examination in Neurodegenerative Diseases of the Brain

**DOI:** 10.1002/alz.092142

**Published:** 2025-01-09

**Authors:** Umur A. Kayabasi

**Affiliations:** ^1^ D‐ NO Visual Therapy Center, Istanbul, Turkey; Uskudar University, İstanbul Turkey

## Abstract

**Background:**

Alzheimer's Disease (AD) is a chronic neurodegenerative disease characterized by loss of memory and cognitive decline associated with an increase in ß‐amyloid (Aß) plaque deposition, neurofibrillary tangle formation (NFT) and inflammation. Fundus Autofluorescein (FAF) can detect lipofuscin secreted by misfolded plaques during the neurodegeneration process. And Optical Scanning Tomography ( OCT ) is able to find out the retinal layers of the accumulation ( inner retinal layers in AD ). lt is also possible to measure macular thickness by OCT ( macular thinning being the earliest sign of AD).

**Method:**

30 patients with PET proven AD were examined by FAF and OCT. Mean age was 71. Also 10 age matched healthy controls underwent retinal exam. 15 AD patients were given Curcumin tablets for 3 days to detect changes after the supplement.

**Result:**

All the patients with AD revealed detectable accumulations by FAF and their location was the inner retinal layers as picked up bt OCT. ln the control group, typical accumulation styles were not seen.

**Conclusion:**

Retinal examination is one of the best methods to detect AD. lt is safe, non‐invasive and reproducable.

